# Assessment of environmental correlates of physical activity: development of a European questionnaire

**DOI:** 10.1186/1479-5868-6-39

**Published:** 2009-07-06

**Authors:** Heleen Spittaels, Charlie Foster, Jean-Michel Oppert, Harry Rutter, Pekka Oja, Michael Sjöström, Ilse De Bourdeaudhuij

**Affiliations:** 1 Department of Movement and Sports Sciences, Ghent University, Watersportlaan 2, 9000 Ghent, Belgium; 2 Department of Public Health, University of Oxford, Old Road Campus, Headington, Oxford, OX3 7LF, UK; 3 Nutritional Epidemiology Unit, UMR INSERM U557/INRA U1125/CNAM/University Paris 13, CRNH IdF, 93017 Bobigny, France; 4 Department of Nutrition, Pitie-Salpetriere Hospital (AP-HP), University Pierre et Marie Curie-Paris6, 75013 Paris, France; 5 National Obesity Observatory, 4150 Chancellor Court, Oxford, OX4 2GX, UK; 6 Urho Kaleva Kekkonen Institute for Health Promotion Research, FIN-33500 Tampere, Finland; 7 Karolinska Institute, Department of Biosciences, Preventive Nutrition, Novum, 141 57 Huddinge, Sweden

## Abstract

**Background:**

Research on the influence of the physical environment on physical activity is rapidly expanding and different measures of environmental perceptions have been developed, mostly in the US and Australia. The purpose of this paper is to (i) provide a literature review of measures of environmental perceptions recently used in European studies and (ii) develop a questionnaire for population monitoring purposes in the European countries.

**Methods:**

This study was done within the framework of the EU-funded project 'Instruments for Assessing Levels of Physical Activity and Fitness (ALPHA)', which aims to propose standardised instruments for physical activity and fitness monitoring across Europe. Quantitative studies published from 1990 up to November 2007 were systematically searched in Pubmed, Web of Science, TRIS and Geobase. In addition a survey was conducted among members of the European network for the promotion of Health-Enhancing Physical Activity (HEPA Europe) and European members of the International Physical Activity and Environment Network (IPEN) to identify published or ongoing studies. Studies were included if they were conducted among European general adult population (18+y) and used a questionnaire to assess perceptions of the physical environment. A consensus meeting with an international expert group was organised to discuss the development of a European environmental questionnaire.

**Results:**

The literature search resulted in 23 European studies, 15 published and 8 unpublished. In these studies, 13 different environmental questionnaires were used. Most of these studies used adapted versions of questionnaires that were developed outside Europe and that focused only on the walkability construct: The Neighborhood Environment Walkability Scale (NEWS), the abbreviated version of the NEWS (ANEWS) and the Neighborhood Quality of Life Study (NQLS) questionnaire have been most commonly used. Based on the results of the literature review and the output of the meeting with international experts, a European environmental questionnaire with 49 items was developed.

**Conclusion:**

There is need for a greater degree of standardization in instruments/methods used to assess environmental correlates of physical activity, taking into account the European-specific situation. A first step in this process is taken by the development of a European environmental questionnaire.

## Background

Despite the numerous health benefits associated with an active lifestyle, the majority of adults in Western countries does not participate in regular physical activities of at least moderate intensity. A survey across member states of the European Union found that about two thirds of adults in these countries does not perform sufficient physical activity for health benefits [1]. For effective interventions, an evidence-based knowledge of physical activity determinants including the environmental ones is essential.

There is increasing interest in comprehensive theoretical frameworks (e.g. ecological models) in which, next to individual, social and cultural factors also physical environmental factors are included [2]. From a public health perspective, research about the influence of the physical or built environment, which is defined as "all of the physical parts of were we live and work (e.g., homes, buildings, streets, open spaces, and infrastructure)" [3], on physical activity appears promising. Indeed, environment-changing interventions have the potential to reach a large proportion of the population as well as to achieve sustainable effects.

Research on the contribution of environmental variables in explaining physical activity behaviour is rapidly expanding. The Active Living Research Network Reference list illustrates this growth, with 101 references in 2004, 160 in 2005 and 301 references in 2006, published in 122 different journals [4]. However, the theoretical gains from such a body of literature have remained modest to date. Bauman re-iterates of this lack of progress, "the plethora of cross sectional analytical papers that show small cross sectional associations ... without really striking gold in terms of identifying the solve-all correlates" [5]. This suggests that measuring environmental determinants is a complex process, both in terms of which environmental variables are relevant to measure as well as how to measure these variables accurately.

Studies of the environment and physical activity have typically used two types of exposure measures, (i) measures of perceptions of the environment using a questionnaire, and (ii) objective measures of the environment derived from observations of the environment (audits, ground truthing) or Geographic Information Systems (GIS) data [6].

Early drafts of measures of perceptions of the environment were criticised for their lack of metric data (e.g. repeatability, face validity) [7]. The development of perceived environmental measures has emerged outside of Europe, either from Australia – in particular the SEID (Social Environmental Individual Determinants) study conducted by Giles-Corti and colleagues [8], or from three research centres in the US (North Carolina – [9]; South Carolina – [10]; California – [11]. As the built environment in Europe differs considerably from those in the US or Australia (e.g. compare the environments of European city centres and those of North American suburbs) this raises questions about the applicability of these questionnaires in a European context. As a consequence a small number of European studies have developed their own questionnaires as part of studies or have adapted international questionnaires to the European context. However, today no consensus exists about which environmental questionnaire should be used in Europe. The latter issue is one of the objectives of an EU-funded project called ALPHA (Instruments for Assessing Levels of Physical Activity and Fitness), that will propose standardised instruments for physical activity and fitness monitoring across Europe [12]. Thus the first objective of this study is to conduct a literature review on currently used questionnaires to assess environmental aspects of physical activity in the general population in Europe. This paper presents the results of this review and based on it proposes a environmental questionnaire for population monitoring purposes in European countries.

## Methods

### PHASE I: Literature review

#### Data sources

An extensive and systematic literature search was conducted to identify currently used questionnaires to assess environmental aspects of physical activity in Europe using the online databases PubMed, Web of Sciences, Transportation Research Information System (TRIS) and Geobase. The search strategy was based on those of Wendel-Vos and colleagues [13], including physical activity-related keywords as physical (in)activity, walking, bicycling, sports, active transportation and environmental-related keywords as physical environment, environmental influence, built environment and environment perception. The search strategy was initially developed in Pubmed and tailored for use in other databases. The search was also restricted to human studies published in English between 1 January 1990 and 30 November 2007. Furthermore, reference lists of relevant publications that were found were examined.

In addition to the systematic literature search we also conducted a survey among the members of the European network for the promotion of Health-Enhancing Physical Activity (HEPA Europe) and European members of the International Physical Activity & the Environment Network (IPEN). We contacted all key expert authors within Europe and asked for details of published or ongoing studies using perceived measures of the physical environment in relation to physical activity.

#### Data Extraction

Studies were included if they met following inclusion criteria (i) reporting measuring the association between a physical activity behaviour and an aspect of the environment using a specific perceived environmental measure. (ii) reporting the metrics of a perceived environmental measure (iii) being from European origin (iv) conducted in the general adult population, 18 years and older without any specific diseases.

Studies were excluded if they were narrative or focused only on the social, political or economical environment or measured the objective environment instead of perceptions. Primary papers found by the literature search were first independently scanned on title and abstract to check whether they met the inclusion criteria by two reviewers (HS, CF). After the initial screening of the studies on title and abstract, full text of the selected papers were retrieved and scanned again. Finally the (un)published papers, abstracts or theses retrieved from the HEPA-Europe and the IPEN networks were also screened. Disagreements were resolved by discussion with a third party (IB).

### PHASE II. Designing a European environmental perceptions questionnaire

Similar to the development of both long and short forms of the International Physical Activity Questionnaire (IPAQ) [14], we aimed at designing a long form of the environmental questionnaire for research purposes and a short form for monitoring purposes.

We did not aim to develop an entirely new questionnaire including a list of new items, but selected themes and items that were already used in other questionnaires. The development process consisted of two steps: (1) selecting the themes and (2) selecting the items. To select the themes we used the results of the literature review to identify the key questionnaires that were used most frequently in Europe. Then, common themes between environmental items in these instruments were analysed and grouped together by themes (such as housing type, access to services, and provision for walking and cycling), each of which was considered for inclusion in the final version of the questionnaire.

To select the items a factor analysis was carried out on data on perceptions of the environment collected in one published European study [15] to identify the highest loading items. Items with factor loading above 0.70 were considered for inclusion in the European questionnaire.

Next, a consensus meeting with an international expert group [see Additional file 1] was organised and all items of both forms of the questionnaire were discussed until consensus was reached on which should be included in the final version

## Results

### Literature search

**Figure 1:**
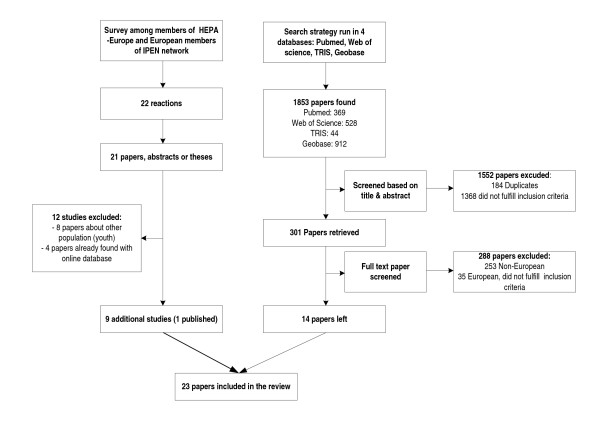
**Flow chart of study selection process**. HEPA-Europe: European Network for the Promotion of Health-Enhancing Physical Activity; IPEN: the International Physical Activity and Environment Network; TRIS: Transportation Research Information Services

The initial computerised literature search resulted in 1853 studies (see Figure 1). Based on the title or the abstract, 1368 papers did not fulfil the inclusion criteria and were excluded together with 184 duplicates. The remaining 301 papers were retrieved and scanned against the inclusion criteria. At this step, 288 papers were excluded (most of them (253) were non-European papers) resulting in 14 papers [15-28]. The reference search did not result in further papers fulfilling the inclusion criteria.

Contacting the members of the HEPA Europe and the IPEN networks yielded twenty papers, abstracts or theses of recent or ongoing studies and resulted in nine additional studies that were not detected by the online literature search, eight unpublished and one published study([29-32,21]; Chaix (personal communication); Davey (personal communication); Trayers (personal communication); Van Keulen (personal communication)).

Thus, this systematic review on currently used environmental questionnaires in Europe identified 23 studies that fully met the inclusion criteria.

### General characteristics of the studies

All 23 studies are summarized in Table 1. Of the 23 detected studies, 15 had been published at the time of the data collection exercise and eight had not. Eight of the 15 published papers were published in the last two years. Nine of the 23 studies were carried out in the United Kingdom, two each in Belgium and Austria, one each in Germany, Sweden, Turkey, Portugal, France, Denmark and the Netherlands, and three studies in two or more EU countries. Study population size varied from 98 to 16230 participants. In 16 of the studies both female as male adults were included, in two studies the participants were university students, in two studies participants were elderly women and men and one study included only female participants. In total 13 different questionnaires were used; the number of the environmental items in these questionnaires varied from two to 108.

**Table 1:** Key characteristics of the studies included in the systematic review, ordered by publication date

**Author**	**Year**	**Country**	**Subjects**	**Questionnaire/kind of items**	**# environmental items**
Rutten et al.	2001	Germany	3343 adults	MAREPS - Local opportunity scale	3

Stahl et al.	2002	Germany/finland	1802 adults	MAREPS - Local opportunity scale	3

De Bourdeaudhuij et al	2003	Belgium	521 adults	NEWS	81

Foster et al.	2004	UK	4265 adults	A4L	4

Rutten et al.	2004	European Union	16 230 EU-residents, 15+	Eurobarometer - Local opportunity scale	3

De Bourdeaudhuij et al	2005	Belgium/Portugal	526 adults	NEWS	81

Alexander et al.	2006	Sweden	98 Swedish adults	IPAQ-E	17

Titze et al.	2005	Austria	509 female runners	Environmental factors	5

Daskapan et al.	2006	Turkey	352 University students	Perceived barriers, lack of resource	2

Poortinga	2006	UK	14 836 adults	Perceived aspects of the social and physical environment	7

Dawson et al.	2007	UK	750 adults	A4L	9

Dawson et al.	2007	UK	680 adults	A4L	9

Harrison et al.	2007	UK	15461	Perceptions of neighbourhood	10

Mota et al.	2007	Portugal	181 elderly	IPAQE – adapted	9

Titze et al.	2007	Austria	634 University students	C4T	18

Chaix B.	unpublished	France	French adults	RECORD	14

Davey R	unpublished	UK	Community-based	ANEWS	54

De Geus B	Doctoral Thesis	Belgium	343 adults	ANEWS – adapted	54

Mygind O	Master Thesis	Denmark	226 adults	ANEWS – adapted	54

Ogilvie D	Doctoral Thesis	UK	Not mentioned	PLE	14

Trayers T	unpublished	UK	Elderly	NEWS – adapted	66

Van Keulen H	unpublished	Netherlands	Not mentioned	NQLS	108

Wright A	abstract	UK	79 Scottish adults	NEWS – adapted	60

Note: MAREPS: a Methodology for the Analysis of Rationality and Effectiveness of Prevention and Health Promotion Strategies; A4L: Active for Life; NEWS: Neighborhood Environment Walkability Scale; IPAQ-E: Environmental module of International Physical Activity Questionnaire; PLE: Perceptions of Local Environment; C4T: Cycling for Transport; NQLS: Neighborhood Quality of Life Study; RECORD: questionnaire Residential Environment and Coronary Disease

### Measures of environmental perceptions

Table 2 presents details for the measures of environmental perceptions used most frequently within a European context. The table also includes details of published data on the metrics of each measure and the definitions and criteria used for scale within each measure.

**Table 2:** Summary descriptive table of environmental questionnaires

**Measure/Country of origin**	**Type and number of environmental items**	**Physical activity**	**Population samples used with measure**	**Assessment of metrics of measures & Scale**	**Findings/Notes**
Neighborhood Environment Walkability Scale (NEWS) (NQLS) USA	Original NEWS (98 items) assessed perceptions of residential density (house type), land use density (walk access to grocery stores, video stores, post offices and fast food restaurants), land use mix-access (shops and parking available locally), street connectivity, walking/cycling facilities Self report via mail	Walking – NEWS Global measure of physical activity plus domains – NQLS	USA and number of international studies for adults	NEWS item scores ranged from 0.58–0.80 (Saelens et al, 2003) Intra class correlations for NEWS ranged from 0.41–0.93 (Brownson et al, 2004) NEWS possessed adequate levels of factorial and criterion validity (Cerin et al, 2006) Scale defined in different dimensions - by time to walk to - 10–15 minute walk - neighbourhood	Papers hint at considerable differences which emerged when the measure was applied to urban and rural participants NEWS is a popular measure of walkability – unsure of its applicability in Europe

Neighborhood Environment Walkability Scale (ANEWS) Shorter version USA	Shortened version (49 items) assessed perceptions of residential density (house type), land use density (walk access to grocery stores, video stores, post offices and fast food restaurants), land use mix-access (shops and parking available locally), street connectivity, walking/cycling facilities Self report via mail	Walking – NEWS	USA and number of international studies for adults	NEWS-A possessed adequate levels of factorial and criterion validity (Cerin et al, 2006) Scale defined in different dimensions - by time to walk to - 10–15 minute walk - neighbourhood - within a 5 minute drive or a 10 minute walk from work or home	As above

IPAQ environmental module (IPAQE) USA then International	This measure (17 items) has 7 core, 4 recommended and 6 optional items, covering similar items to NEWS and NQLS. Also included walking access to a public transport transit service, include cost and low cost to access to places to be active and seeing other active in neighbourhood Self report via mail or telephone	Global measure of physical activity	Random sample of Swedish adults (IPS)	Test-retest reliability testing with 98 Swedish adults ICC: 0.41–0.96 (men) 0.27–0.98 (women) Scale Neighborhood, easy walking distance with 10–15 mins	

Cycling for Transport (C4T) Austria	This measure (25 items) assessed perceived environmental attributes of cycling for transportation. This includes functional, safety, aesthetics, destination and home neighbourhood features Self report via mail	Cycling	Sample of Suisse students	- Factor analysis, - Test-retest reliability testing with 32 students Scale – on journey to and from university	- Reduced 25 to 23 items, (6 factors + 5 single items) Cronbach α: 0.39–0.66 -Agreement 0.62–0.89

Perceptions of local environment (PLE) UK Awaiting assessment	This measure (16 items) assessed perceptions of the local area and access to personal transport Self report via mail	Walking and cycling	1322 households in Glasgow	Test – retest reliability (N = 23) Scale in my local area	0.55–0.89 (one item 0.21)

Active 4 Life (A4L) perceptions of environment and walking UK	This measure (10 items) assessed access to green space, local shops, safety (day and night), Aesthetics, traffic, access to sports facilities and social support Self report via face to face interviews	Walking	Random sample of UK adults aged 16–74 years	No reported assessment of validity or reliability. Scale defined by neighbourhood	Response to this measure was differentiated by gender

Residential Environment and Coronary Disease (RECORD) France	This measure (14 items) assessed availability to local facilities, places for walking, aesthetics, safety from traffic and safety from crime. All items were formulated negatively. Self report via mail	Walking & sport			

The Neighborhood Environment Walkability Scale (NEWS) and the abbreviated version of the NEWS (ANEWS) and the Neighbourhood Quality of Life Study (NQLS) have been most commonly used (eight times). NEWS has been used in a number European studies in conjunction with IPAQ. Most studies reported some adaptation of the NEWS items in terms of language and readability. Sometimes NEWS items that were considered unsuitable for the European context were removed and sometimes other items were added. The NEWS was assessed for its metrics in the US, Australia and Belgium and it appeared to have adequate metric properties [11,33-35,15] and had adequate correlations with objective assessments of environments [11,34].

The IPAQ Environmental module (IPAQE) has been used less extensively (two studies) but is closely aligned with the popular IPAQ measure of physical activity. IPAQE was developed by the members of the IPAQ core group [21] and reflects current opinions and experiences on environmental correlates. The measure was developed in conjunction with IPAQ as a tool for population monitoring. The questionnaire can be administered via the mail or telephone.

The Cycling for Transport (C4T) measure was used in an Austrian study of environmental, social and personal correlates of cycling for transportation for university students [28]. The measure was developed using a review of environmental correlates of cycling and subsequent focus group discussions among student cyclists. This measure is the only specific measure for cycling.

The Perceptions of Local Environment (PLE) measure was used as part of Dr David Ogilvie's PhD thesis, looking at perceptions of the environment in relation to active travel. The measure has been assessed for test-retest reliability and is clearly applicable in the UK [31].

The Active for Life (A4L) measure was used as part of a study looking at perceptions of the environment in relation to walking with a sample of adults across England [19,36]. The measure is specific to walking but has not yet been assessed for its metrics.

The questionnaire Residential Environment and Coronary Disease (RECORD) was developed recently by Basile Chaix in France and assessed aspects of physical activity and of the related residential environment. The questionnaire also includes specific aspects of the social environment but has not yet been assessed for its metrics.

### Criteria for perceived environmental measure for Europe

The ideal European perceived environmental measure should be able: (1) to be applicable to the European context across wide range of different environmental contexts and behaviour patterns; (2) to be comparable across European data sets; (3) to have clearly defined neighbourhood and area properties, cogent with resident's definitions; (4) to be comparable with objective measures of the environment as related to physical activity; (5) to have established metric properties (temporality, face validity, repeatability); (6) to relate specific environmental items to specific physical activities, particularly walking and cycling for leisure and transport; (7) to be easy to administer by mail, telephone or face to face.

Not surprisingly, none of the eight key questionnaires met all of the above criteria. Therefore, we developed an environmental questionnaire specific for the European context.

### Designing a European environmental questionnaire

The first step in designing a European environmental measure was to select the themes that should be asked for. This was done by analysing the common themes between environmental items in the eight key questionnaires. The table in the additional file 2 [see additional file 2] presents the full list of items from all eight measures, categorised per theme. The measures have very similar clusters of environmental themes, particularly NEWS, ANEWS, and NQLS (as they are modifications of the same base questionnaire). These common themes are: (1) housing types; (2) local facilities; (3) access to services; (4) street connectivity; (5) places for walking and cycling; (6) neighbourhood surroundings/aesthetics; (7) safety from traffic; (8) safety from crime.

The shorter measures (IPAQE, C4T, A4L, PLE, and RECORD) also include items related to other themes. In addition, NEWS asked questions on perceived satisfaction with the neighbourhood levels of facilities, crime, safety, services, connectivity, and aesthetics. NQLS included items related to physical activity opportunities and exercise equipment at home and within the local environment. It also covered aspects of social cohesion and social capital. RECORD also assessed social cohesion. One item unique to IPAQE and PLE was the number of motor vehicles available in the household. RECORD was the only questionnaire that included an item about quality of sports equipments and one item about vandalism and graffiti.

After identifying the common themes, nine of them were selected for the questionnaire (both long and short form version), taking into account the guidelines mentioned earlier. The selected themes were: (1) types of residences in your neighbourhood,(2) distances to local facilities, (3) walking or cycle infrastructure in your neighbourhood, (4) maintenance of infrastructure in your neighbourhood, (5) neighbourhood safety, (6) how pleasant is your neighbourhood, (7) cycling and walking network, (8) home environment, (9) workplace or study environment.

A second step was to select the items for each theme. As the NEWS questionnaire was one of the most commonly used measures, factor analysis was done on NEWS data obtained from a previous study in Belgium [15]. Based on these results, the items with high factor loadings (>0.70) were selected for the questionnaire, e.g.: ' stores are within easy walking distance of my home' 'There are sidewalks in most of the streets in my neighbourhood' 'The sidewalks in my neighbourhood are well maintained' ' The crime rate in my neighbourhood makes it unsafe to go on walks at night.'.

If applicable, NEWS items were included in their original form, making it possible to compare future datasets with international studies using the NEWS questionnaire. A draft questionnaire was constructed following discussions between all authors of this manuscript, and a consensus meeting with an international expert group was then organised to make a final selection of items. This comprised nine themes with a total of 49 items for the long form [see additional file 3]. For the short form of the questionnaire [see additional file 4] the number of items was reduced to 11 items, but a minimum one item was included within each theme.

## Discussion

The main purpose of this study was to conduct a systematic overview of perceived environmental measures in relation to physical activity that are currently used in Europe in order to develop a questionnaire for population monitoring purposes in the EU member states. In total 23 published or unpublished European studies were identified by literature search. This is a small number compared with the increasing number of international environmental studies (mostly from the US and Australia) that have been conducted in recent years. This and the fact that most of the studies have been published very recently or are still underway, indicates that research about the influence of the physical environment on physical activity is still in its infancy in Europe.

There were eight key environmental questionnaires that have been identified in the European literature. The NEWS, ANEWS and the NQLS have been most commonly used. However, the NEWS and its modified versions were all developed and tested on their metric properties outside Europe. Most European authors who used these questionnaires in their studies reported some adaptations of the NEWS items both in terms of language and readability as in removing unsuitable items or adding new ones. Consequently, this raises questions about the appropriateness of the use of the original NEWS in a European context. Other authors also developed their own (shorter) questionnaire introducing new items. So, similar with international literature [37] there is inconsistency in measuring the perceived environment in Europe and is it difficult to make inter-study comparisons. Thus there is a need for a greater degree of standardization in perceived environmental measures.

As none of the identified key questionnaires comply with all the desired elements of an ideal European environmental questionnaire, steps were taken to design a European instrument. Two versions (long and short) of a European environmental questionnaire were designed taking into account the earlier mentioned guidelines:

(1) The European instrument was specifically designed for a European situation, including items of key questionnaires designed in Europe. To increase the comparability with international studies, NEWS items were also included on the condition that they were applicable within the European context. Future research should investigate whether this questionnaire represents an appropriate instrument for assessing perceptions of the environment in all parts of Europe, and indeed whether or not a standardised instrument is possible at all. It is, for example, very difficult to measure perceptions of safety across Europe as the ways in which people perceive safety are influenced by many factors, including social and cultural norms, and previous personal experiences, that vary greatly both between and within countries.

(2) The long IPAQ has been thoroughly tested, and validated against objective assessment of physical activity (accelerometry) [14,38], and has recently been used (in a modified version) for measuring physical activity in European populations [39,40]. Most of the other measurements discussed in this paper focus on transport-related physical activity (usually walking) and leisure time physical activity (usually walking, sometimes cycling or sports), and only NQLS assesses the domain of physical activity at home. Except for the study by de Geus et al. [29], none of the reviewed questionnaires attempted to assess environmental items in relation to work. In the study of de Geus et al. some questions were added to the NEWS questionnaire including "destinations to work", "facilities for cyclists at the workplace" and "traffic variables on the road to work". However, the European instrument now includes subscales related to all the four physical activity domains i.e. transport-related physical activity, physical activity at work, physical activity at home and leisure time physical activity.

(3) Another issue that increases the inconsistency in measurements is the lack of standardisation in neighbourhood definitions. These are ranging from vague formulations as 'neighbourhood' and 'local area' to more specific definitions 'within a 5 to 10 minute walk'. In the European questionnaire, the following definition of neighbourhood is used: "By your neighbourhood we mean the area ALL around your home that you could walk to in 10–15 minutes – approx 1.5 km" (or "1 mile" for UK-context).

(4) (5) As Bauman [5] already noted there is a need for improved and more sophisticated exposure measures (perceived and objective), and better assessment of walking and related behaviours. Only one of the reviewed studies compared the assessment of perceptions with objective measurements [15]. In the international literature the number of such studies is also limited [13]. However some studies have shown that for example NEWS has adequate correlations with objective assessments of environments [11,34]. A positive finding is that the questionnaires that are developed inside Europe have shown good metric properties. More research is needed to confirm if the European questionnaire is comparable with objective measures and if the English and translated versions of the questionnaire have valid metric properties in their relevant countries.

(6) In the European instrument items were included to assess the influence of the physical environment on not only walking but also on cycling behaviour. In contrast with the US and Australia, cycling is a prevalent physical activity in many European countries, both as leisure physical activity [41] and as transport-related behaviour [42] and therefore measuring cycling infrastructure is very relevant in European studies.

(7) Most of the existing measurements have been administered by mail which is the most feasible type of administration in population monitoring. Nevertheless the length of some measurements is too long for monitoring (e.g. NQLS 108 items, NEWS, 98 items), especially knowing that the environmental measurement will be part of a broader (physical activity) assessment. Therefore, efforts were done to design a shorter questionnaire. The long version of the European instrument counts 49 items, which seems feasible for research purposes. Further, a short form of 11 items was developed for monitoring purposes.

## Conclusion

Most of the identified European studies used adapted versions of questionnaires that were developed outside Europe and focused only on walkability. There is need for a greater degree of standardization in measurements of the perceived environment, taking into account the European-specific situation, and including the influence of the physical environment on cycling behaviour. On behalf of the ALPHA project a first step of standardization was taken: the authors of this review, together with an international expert group, developed an environmental questionnaire specifically for use within the European context. Two versions were developed: a long version for research purposes [see additional file 3] and a short version for monitoring purposes [see additional file 4]. Future research is needed to test this questionnaire for reliability and validity in different languages and in different European countries.

## Competing interests

The authors declare that they have no competing interests.

## Authors' contributions

MS, PO, IDB, HR and JMO identified the research question and design of this study as part of the ALPHA project. HS led the literature review and designed the search strategy. HS and CF carried out the literature searches and screened the initial results, extracted data, analysed the findings, drafted the tables and the manuscript. HS, CF, HR, PO, JMP and IDB developed a first draft of the environmental questionnaire. All authors participated in the expert meeting, contributed to synthesising the results and critical revision of the manuscript, and approved the final version.

## Supplementary Material

Additional file 1 **International expert group**. List of the members of the international expert group. Click here for file

Additional file 2 **Items for eight questionnaires in relation to physical activity used in Europe**. Table presenting the full list of items from all eight questionnaires, categorised per theme. Click here for file

Additional file 3 **Long measure of environmental perceptions: active travel and physical activity**. ALPHA environmental questionnaire (long form) in English. Click here for file

Additional file 4 **Short measure of environmental perceptions: active travel and physical activity**. ALPHA environmental questionnaire (short form) in English. Click here for file
