# Supplementary material for: Assessment of environmental correlates of physical activity: development of a European questionnaire
Source: Int J Behav Nutr Phys Act. 2009 Jul 6;6:39. doi: 10.1186/1479-5868-6-39 (PMC2713198; doi:10.1186/1479-5868-6-39)
Supplement: Additional file 1 — International expert group. List of the members of the international expert group. [file 1479-5868-6-39-S1.pdf]

<u>International expert group</u>

- James F Sallis (US)
- Neville Owen (Australia)
- Klaus Gebel (Germany)
- Fiona C.L. Bull (UK)
- Christopher Gidlow (UK)
- Basile Chaix (France)
- David Ogilvie (UK)
- Sylvia Titze (Austria)
- Frank Van Lenthe (Netherlands)
- Patrick Bergman (Sweden)
- Kristina Sundquist (Sweden)
- Melvin Hillsdon (UK)
- Roger Macket (UK)
- Andrea Backovic (Slovenia)
