# Supplementary material for: Assessment of environmental correlates of physical activity: development of a European questionnaire
Source: Int J Behav Nutr Phys Act. 2009 Jul 6;6:39. doi: 10.1186/1479-5868-6-39 (PMC2713198; doi:10.1186/1479-5868-6-39)
Supplement: Additional file 2 — Items for eight questionnaires in relation to physical activity used in Europe. Table presenting the full list of items from all eight questionnaires, categorised per theme. [file 1479-5868-6-39-S2.pdf]

Items for eight environmental questionnaires in relation to physical activity used in Europe

	NEWS	ANEWS	NQLS	IPAQE	A4L	C4T	PLE	RECORD
**Items**	83 items	54 items	106 items	17items	9 items	23 items	17 items	14 items
**Housing types**								
Detached single-family residences	X	X	X					
Townhouses or rows houses of 1-3 stories	X	X	X					
Apartments or condos 1-3 stories	X	X	X					
Apartments or condos 4-6 stories	X	X	X					
Apartments or condos 7-12 stories	X	X	X					
Apartments or condos more than 13 stories	X	X	X					
Mix of single-family residences and townhouses, row houses, apartments or condos								
What is the main type of housing in your neighbourhood				X				
**Local facilities (shops, exercise facilities, parks)**								
Grocery store	X	X	X		X (local shops)	X (along the route)		
Supermarket	X	X	X					
Hardware store	X	X	X					
Fruit/vegetable market	X	X	X					
Laundry/dry cleaners	X	X	X					
Clothing store	X	X	X					
Post office	X	X	X					
Library	X	X	X					
Elementary school	X	X	X					
Other schools	X	X	X					
Book store	X	X	X					
Fast food restaurant	X	X	X					
Coffee place	X	X	X					
Bank/credit union	X	X	X					
Non-fast food restaurant	X	X	X					
Video store	X	X	X					
Pharmacy/drug store	X	X	X					
Salon/barber shop	X	X	X					
Your job or school	X	X	X					
Bus or trolley stop / lack of..	X	X	X				X	X
Park/ lack of..	X	X	X	X (free or low cost recreation facilities)	X		X	
Recreation center/ Lack of..	X	X	X		X (leisure centre)			X
Gym of fitness facility/ Lack of.	X	X	X					X
Lack of maintenance of collective equipments								X
Lack of quality of sportsequipments								X
**Access to services**								
Possible to do most shopping at local stores	X		X					
Stores are within easy walking distance	X	X	X	X			X (too far)	
Parking is difficult in local shopping areas	X	X (single item)	X					
There are many places to go within easy walking distance	X	X	X	X (Optional)				
It is easy to walk to a transit stop	X	X	X	X				
The streets are hilly, difficult to walk/ to bicycle	X	X (single item)	X			X		
There are many canyons, hillsides in my neighbourhood that limit the number of routes	X	X (single item)	X					

for getting from place to place		Major barriers in stead of canyons,…						
**Street connectivity**								
Streets do no have many cul-de-sacs	X	X (single item)	X					
There are walkways that connect cul-de-sacs to streets, trails, or other cul-de-sacs	X		X					
Distance between intersections is usually short	X	X	X					
There are many four-way intersections	X		X	X (Optional)				
There are many alternative routes	X	X	X					
Easier to take shortcuts with the bicycle than with a car						X		
Cycling is faster compared to driving a car						X		
**Places for walking and cycling**								
There are sidewalks on most of the streets	X	X	X	X				
Sidewalks are well maintained	X		X	X (Optional)				
There are bicycle or pedestrian trails/convenient routes for walking	X		X	X			X	
Sidewalks are separated from the road, by parked cars	X	X	X					
Sidewalks are separated from the road, by grass/dirt strip	X	X	X					
Streets are well lit at night	X (safety from crime)	X	X			X		
Walkers and bikers can by easily seen by people in their homes	X (safety from crime)	X	X					
There are crosswalks and pedestrian signals to help walkers cross busy streets	X (safety from traffic)	X	X (neighbour hood safety)					
It is safe to ride a bike			X				X (dangero us)	
Places for bicycling are well maintained and not obstructed/convenient routes for cycling				X (optional)			X	
Parking facilities for bikes						X		
It is pleasant to walk/unpleasant							X	X
**Neighbourhood surroundings/Aesthetics**								
There are trees along the streets	X	X	X		X (high / low aesthetic s)	X	X (unattrac tive)	
Trees give shade for the sidewalks	X		X					
There are many interesting things to look at while walking	X	X	X	X (recommen ded)		X		
Neighbourhood is generally free from litter/ has lot of litter	X		X					X
There are many attractive natural sights	X	X	X					
There are (un) attractive buildings/homes	X	X	X			X		X
High air pollution						X		X
There is lot of traffic noise						X	X	
Little green space							X	X
Lots of noise from people living in the neighbourhood								X

**Safety from traffic**								
So much traffic along the street I live on that it makes it difficult or unpleasant to walk	X		X	X (recommended)	X (high/low traffic levels)			
So much traffic along nearby streets that it makes it difficult or unpleasant to walk	X	X	X					
Speed of traffic on the street I live on is usually slow	X		X					
Speed of traffic on most nearby streets is usually slow	X	X	X					
Most drivers exceed the posted speed limits while driving	X	X	X					
Crosswalks help walkers feel safe crossing busy streets	X		X					
When walking there are a lot of exhaust fumes	X		X					
So much traffic on the streets that it make it difficult or unpleasant to ride a bicycle / substantial detours are necessary				X (Optional)		X		
The percentage of routes with little traffic is…						X		
Cycling is unsafe because of high traffic						X		
Busy crossings without traffic lights						X		
Cycling is unsafe because of tramway lines						X		
There are many traffic lights						X		
It is impossible to bike continuously with almost no stops						X		
There is little traffic/to much traffic							X	X
It is safe to cross the road							X	
**Safety from crime**								
I see and speak to other people when I am walking	X		X					
There is a high crime rate	X	X	X					
The crime rate makes it unsafe to go on walks during the day	X	X	X	X (optional)	X			
The crime rate makes it unsafe to go on walks at night	X	X	X	X	X		X	
My neighbourhood is safe enough so that I would let a 10-year-old boy walk around my block alone in the daytime			X					
There is a high likelihood of bikes being stolen at university site						X		
I wouldn't like to leave my bike in front of my residence because of the risk of theft						X		
Cyclists often have disputes with car-drivers						X		
Cyclists often have disputes with pedestrians						X		
Cyclists often have disputes with cyclists						X		
People are likely to be attacked							X	
Vandalism, graffiti on the walls								X
Rudeness, aggressive attitude to certain people								X

**Satisfaction with neighbourhood. How satisfied are you with…**								
Number of pedestrian cross-walks	X							
The highway access from your home	X							
The access to public transportation	X							
Your commuting time to work/school	X							
The asses to shopping	X							
How many friends you have	X							
The number of people you know	X							
How easy and pleasant it is to walk	X							
How easy and pleasant it is to bicycle	X							
The quality of schools	X							
Access to entertainment	X							
The safety from threat or crime	X							
The amount and speed f traffic	X							
The number and quality of food stores	X							
The number and quality of restaurants	X							
Your neighbourhood as a good place to raise children	X							
Your neighbourhood as a good place to live	X							
How you feel about living in your local area							X	
**Social support/Modelling**								
Has Company					X			
Prefers company					X			
I see many people being physically active				X (recommended)				
**Home environment**								
Stationary aerobic equipment			X					
Bicycle			X				X	
Dog			X					
Trampoline for jogging in place			X					
Running shoes			X					
Swimming pool			X					
Weight lifting equipment			X					
Skis (snow or water)			X					
Toning devices			X					
Aerobic workout video or audiotapes			X					
Step aerobics, slide aerobics			X					
Skates			X					
Sports equipment			X					
Surf board, boogie board, windsurf board			X					
Canoe, row boat kayak			X					
How many motor vehicles in working order are there at your household				X (recommended)			X	
**Convenient Facilities**								
Aerobic dance studio			X					
Basketball court			X					
Beach, lake, river, or creek			X					
Bike lane or trails			X					
Golf course			X					
Health spa/gym			X					
Martial arts studio			X					
Playing field			X					

Public park			X					
Public recreation center			X					
Racquetball/squash court			X					
Running track			X					
Skating rink			X					
Sporting goods store			X					
Swimming pool			X					
Walking/hiking trails			X					
Tennis courts			X					
Dance studio			X					
**Social cohesion/capital**								
People around my neighbourhood are willing to help their neighbours			X					
This is a close-knit neighbourhood			X					
People in this neighbourhood can be trusted			X					
People in this neighbourhood generally don’t get along with each other			X					
People in this neighbourhood do not share the same values			X					
