# Supplementary material for: Assessment of environmental correlates of physical activity: development of a European questionnaire
Source: Int J Behav Nutr Phys Act. 2009 Jul 6;6:39. doi: 10.1186/1479-5868-6-39 (PMC2713198; doi:10.1186/1479-5868-6-39)
Supplement: Additional file 3 — Long measure of environmental perceptions: active travel and physical activity. ALPHA environmental questionnaire (long form) in English. [file 1479-5868-6-39-S3.pdf]

Project ALPHA

Long measure of environmental perceptions: active travel and physical activity

We would like to find out more information about the way that you perceive or think about your neighbourhood. Please answer as honestly and completely as possible and provide only one answer for each item. There are no right or wrong answers and your information will be kept confidential.

1. Types of residences in your neighbourhood

How common are following types of residences in your immediate neighbourhood?

By your neighbourhood we mean the area ALL around your home that you could walk to in 10-15 minutes - approx 1 mile or 1.5 km

Please put one check mark (✓) per answer that best applies to you and your view of your neighbourhood (

	None	A few	Some	Most	All
a) Detached houses					
b) Semi-detached townhouses, terraced houses					
c) Flats of 6 floors or more					

2. Distance to local facilities

About how long would it take to get from your home to the *nearest* businesses or facilities listed below if you WALKED to them?
Please put one check mark (✓) for each business or facility.

The nearest…	1-5 min	6-10 min	11-20 min	21-30 min	>30 min
a) Local shop: grocery shop, bakery, butcher etc.					
b) Supermarket					
c) Local services such as a bank, post office or library, …					
d) Restaurant, café, pub or bar					
e) Fast-food restaurant or takeaway					
f) Bus stop, tram, metro or train station					
g) Sport and leisure facility such as a swimming pool, sports field or fitness centre					
h) Open recreation area such as a park, beach or other open space					

3. Walking or cycling infrastructure in your neighbourhood

By your neighbourhood we mean the area ALL around your home that you could walk to in 10-15 minutes - approx 1 mile or 1.5 km

Please circle one answer per statement

	Strongly disagree	Somewhat disagree	Somewhat agree	Strongly agree	I don't know
a) There are special lanes, routes or paths to cycle in my neighbourhood	1	2	3	4	5
b) There are traffic-free cycle routes in my neighbourhood	1	2	3	4	5
c) There are sidewalks in my neighbourhood	1	2	3	4	5
d) There are pedestrian zones in my neighbourhood for shopping	1	2	3	4	5

4. Maintenance of infrastructure in your neighbourhood

By your neighbourhood we mean the area ALL around your home that you could walk to in 10-15 minutes - approx 1 mile or 1.5 km

Please circle one answer per statement

	Strongly disagree	Somewhat disagree	Somewhat agree	Strongly agree	Don' t know/ not applicable
a) The cycling paths in my neighbourhood are well maintained	1	2	3	4	5
b) The sidewalks in my neighbourhood are well maintained	1	2	3	4	5
c) The public recreation facilities, parks or other open spaces in my neighbourhood are well maintained	1	2	3	4	5

5. Neighbourhood safety

By your neighbourhood we mean the area ALL around your home that you could walk to in 10-15 minutes - approx 1 mile or 1.5 km

Please circle one answer per statement

	Strongly disagree	Somewhat disagree	Somewhat agree	Strongly agree
a) It is not safe to leave a bicycle locked in my neighbourhood	1	2	3	4
b) There are not enough safe places to cross busy streets in my neighbourhood	1	2	3	4
c) Walking is unsafe because of the traffic in my neighbourhood	1	2	3	4
d) Cycling is unsafe because of the traffic in my neighbourhood	1	2	3	4
e) It is unsafe in my neighbourhood during the day because of the level of crime	1	2	3	4
f) It is unsafe in my neighbourhood during the night because of the level of crime	1	2	3	4

6. How pleasant is your neighbourhood?

By your neighbourhood we mean the area ALL around your home that you could walk to in 10-15 minutes - approx 1 mile or 1.5 km)

Please circle one answer per statement

	Strongly disagree	Somewhat disagree	Somewhat agree	Strongly agree
a) My local neighbourhood is a pleasant environment for walking and cycling	1	2	3	4
b) My neighbourhood is generally free from litter or graffiti	1	2	3	4
c) There are trees along the streets in my neighbourhood	1	2	3	4
d) In my neighbourhood there are a lot of badly maintained, unoccupied or ugly buildings	1	2	3	4

7. Cycling and walking network

By your neighbourhood we mean the area ALL around your home that you could walk to in 10-15 minutes - approx 1 mile or 1.5 km

Please circle one answer per statement

	Strongly disagree	Somewhat disagree	Somewhat agree	Strongly agree	I don't know
a) Cycling is quicker than driving in my neighbourhood during the day	1	2	3	4	5
b) It is easier to take shortcuts with a bicycle or walking than with a car	1	2	3	4	5
c) There are many intersections in my neighbourhood	1	2	3	4	5
d) There are many different routes for cycling or walking from place to place in my neighbourhood so I don't have to go the same way every time	1	2	3	4	5

8. Home Environment

	*Please tick **Yes** or **No*** [✓]	
	**Yes**	**No**
a) Do you have a bicycle for your personal use?		
b) Do you have a garden? (e.g. yard, allotment or city garden)?		
c) Do you have small sports equipment such as a ball, racquets, …for your personal use?		
d) Do you have exercise equipment such as weights, treadmill, stationary cycle, …for your personal use?		
e) Do you have access to a car?		
f) Do you have a dog ?		

9. Workplace or study environment

A. How far do you have to travel to get to your usual place of work or study?

I do not work or study → Please skip part B
I usually work at home or from home → Please skip part B
The distance to my work or place of study is _____________miles/kilometres (circle as appropriate)

B. At your work or place of study do you have….?

	Please tick one box only [✓]	
	**Yes**	**No**
a) …escalators or lifts		
b) …stairs		
c) …fitness centre/equipment		
d) …bicycles provided by employer or school		
e) …a safe place to leave a bike		
f) …a free car park		
g) …showers and changing rooms		
h) …exercise classes (e.g. aerobics classes)		
i) …sports club / association (e.g. running club)		
j) …employer subsidised public transport / cycling		
