# Supplementary material for: Assessment of environmental correlates of physical activity: development of a European questionnaire
Source: Int J Behav Nutr Phys Act. 2009 Jul 6;6:39. doi: 10.1186/1479-5868-6-39 (PMC2713198; doi:10.1186/1479-5868-6-39)
Supplement: Additional file 4 — Short measure of environmental perceptions: active travel and physical activity. ALPHA environmental questionnaire (short form) in English. [file 1479-5868-6-39-S4.pdf]

Assessing Levels of Physical Activity

Project ALPHA

Short measure of environmental perceptions: active travel and physical activity

Please circle the answer that best applies to you and your view of your neighbourhood. By neighbourhood we mean the area ALL around your home that you could walk to in 10-15 minutes - approx 1mile or 1.6 km

	Strongly disagree	Somewhat disagree	Somewhat agree	Strongly agree	
a) Most of the houses in my neighbourhood are detached houses	1	2	3	4	
b) Many shops, stores, markets or other places to buy things I need are within easy walking distance of my home	1	2	3	4	
c) There is a transit stop (such as bus stop, train, trolley or tram station) within easy walking distance of my home	1	2	3	4	
d) There is an open recreation area (e.g. park, beach or other open space) within easy walking distance of my home	1	2	3	4	
e) There are many different routes for cycling or walking from place to place in my neighbourhood so I don't have to go the same way every time	1	2	3	4	
f) Walking and cycling are unsafe because of the traffic in my neighbourhood	1	2	3	4	
g) Walking and cycling are unsafe because of the level of crime in my neighbourhood	1	2	3	4	
h) My local neighbourhood is a pleasant environment for walking and cycling	1	2	3	4	
i) I have access to exercise and sports equipment at home e.g. weights, racquets, skis for personal use	1	2	3	4	
j) My workplace provides facilities to support me walking or cycling to work e.g. changing rooms, bike storage	1	2	3	4	N/A
k) I have access to exercise and sports facilities at work e.g. fitness centre/equipment, stairs	1	2	3	4	N/A
